# Performance of Oriented Strand Board Made of Heat-Treated Bamboo (*Dendrocalamus asper* (Schult.) Backer) Strands

**DOI:** 10.3390/polym16121692

**Published:** 2024-06-14

**Authors:** Sena Maulana, Astri Aulia Suwanda, Rio Ardiansyah Murda, Petar Antov, Rahma Nur Komariah, Muhammad Iqbal Maulana, Sarah Augustina, Seng Hua Lee, Melbi Mahardika, Aditya Rianjanu, Tarmizi Taher, Lubos Kristak, Yazid Bindar, Apri Heri Iswanto, Muhammad Adly Rahandi Lubis

**Affiliations:** 1 Department of Forestry Engineering, Institut Teknologi Sumatera (ITERA), South Lampung, Lampung 35365, Indonesia; sena001@brin.go.id (S.M.); astri.suwanda@rh.itera.ac.id (A.A.S.); rahma_nur_komariah@ymail.com (R.N.K.); 2 Research Center for Biomass and Bioproducts, National Research and Innovation Agency, Cibinong 16911, Indonesia; muha352@brin.go.id (M.I.M.);; 3 Faculty of Forest Industry, University of Forestry, 1797 Sofia, Bulgaria; 4 Department of Wood Industry, Faculty of Applied Sciences, Universiti Teknologi MARA, Cawangan Pahang Kampus Jengka, Bandar Tun Razak 26400, Malaysia; 5 Department of Material Engineering, Institut Teknologi Sumatera (ITERA), South Lampung, Lampung 35365, Indonesia; 6 Center for Green and Sustainable Materials, Institut Teknologi Sumatera (ITERA), South Lampung, Lampung 35365, Indonesia; 7 Department of Environmental Engineering, Institut Teknologi Sumatera (ITERA), South Lampung, Lampung 35365, Indonesia; 8 Faculty of Wood Sciences and Technology, Technical University in Zvolen, 96001 Zvolen, Slovakia; kristak@tuzvo.sk; 9 Department of Chemical Engineering, Faculty of Industrial Technology, Institut Teknologi Bandung, Jl. Ganesha 10, Bandung 40132, Indonesia; 10 Department of Forest Product, Faculty of Forestry, Universitas Sumatera Utara, Padang Bulan, Medan 20155, Indonesia

**Keywords:** bamboo, heat treatment, mechanical properties, oriented strand board, phenol-formaldehyde, physical properties

## Abstract

This study aimed to analyze the effect of pre-heat treatment on bamboo strand properties and its impact on the properties of the resulting bamboo-oriented strand board (BOSB). Giant bamboo (*Dendrocalamus asper* (Schult.) Backer) with a density of 0.53 g cm^−3^ was converted into bamboo strands. These strands were pre-heat-treated at 140 and 160 °C for a duration of 1, 2, and 3 h. Changes in the chemical composition of the strand due to subsequent treatment were assessed. Fourier-transform infrared spectroscopy (FTIR) and X-Ray diffraction analysis (XRD) were used to determine the changes in the chemical composition of bamboo strands. The BOSB panels were produced with a target density of 0.7 g cm^−3^. The manufacturing of the BOSB was conducted in three layers with a ratio of 25:50:25, bonded with phenol-formaldehyde resin. The physical and mechanical properties of the laboratory-fabricated BOSB were tested in compliance with the criteria given in JIS A 5908 standards. Comparisons were made against OSB CSA 0437.0 Grade O-1 commercial standard. The pre-heat treatment led to chemical alterations within the material when set at 140 and 160 °C for 1 to 3 hours (h). FTIR spectral analysis demonstrated that longer exposure and higher temperatures resulted in fewer functional groups within the bamboo strands. The increased temperature and duration of pre-heat treatment enhanced the crystallinity index (CI). The dimensional stability and mechanical properties of the composites were improved significantly as hemicellulose and extractive content were reduced. This study demonstrated that the pre-heat treatment of bamboo strands at a temperature of 160 °C for a duration of 1 h was an adequate approach for heat modification and fabrication of BOSB panels with acceptable properties according to OSB CSA 0437.0 Grade O-1 commercial standard.

## 1. Introduction

Bamboo has gained attention as an environmentally friendly alternative to traditional timber materials used in construction. In recent years, bamboo has gained popularity as a substitute for wood due to its rapid growth, short rotation cycle, simple cultivation methods, excellent mechanical strength, moderate to high density, straightforward processing, and versatility in various industrial applications. Additionally, the global focus on bamboo has significantly risen due to the shortage of forest resources and increased global demand for wood raw materials [1,2]. A viable approach for enhanced valorization of bamboo is its use for manufacturing lignin-based composites, e.g., bamboo oriented strand boards (BOSBs) [3,4,5]. However, there are certain challenges associated with the use of bamboo as the primary raw material for manufacturing BOSBs connected with its high levels of extractive substances [6,7,8,9,10]. These extractives can interfere with properties and durability, ultimately leading to increased production costs.

Previous studies have explored different approaches for modifying bamboo strands, intended for producing BOSBs, including steam treatment [11,12,13], a combination of steam treatment with rinse by 1% NaOH [4,7], steam treatment and physical modification [4,14,15], alkaline immersion treatment [16], as well as post-heat treatment [9,17]. Post-heat treatment of BOSBs resulted in enhanced dimensional stability of the composites but decreased their mechanical properties, possibly related to the increased brittleness of the materials after thermal treatment [9,18]. The main benefits of heat treatment include a decrease in weight, improved dimensional stability, superior weather resistance, and improved color stabilization resulting from the chemical transformation and breakdown of the cell wall constituents. The degradation of hemicelluloses during heat treatment enhances dimensional stability by improving the hydrophobic properties due to an increase in lignin concentration.

According to various studies that have been published [4,9,11,12,13,14,15,17], heat modification has the potential to be used to improve the properties of BOSBs. In general, pre-heat treatment is more prominent compared to post-heat treatment. Nevertheless, the pre-heat treatment utilized in previous research involved the use of steam, which poses a considerable level of operational intricacy. Hence, there is a pressing need for the advancement of a more straightforward pre-heat treatment method that can enhance the quality of OSBs. The use of pre-heat treatment using a standard oven was carried out earlier by Silva et al. [19] on OSB manufacture from *Pinus tadea*. Pre-heat treatment was carried out at temperatures of 160, 180, and 200 °C. However, the resulting BOSB exhibited deteriorated mechanical properties. These findings have sparked curiosity to further explore the effects of pre-heat treatment on the quality and performance of OSBs. Therefore, the aim of this research work was to investigate and evaluate the effect of pre-heat treatment on bamboo strand properties and its impact on the properties of the resulting bamboo (*Dendrocalamus asper* (Schult.) Backer) BOSB. The findings from this research have the potential to be used in establishing sustainability standards for enhanced commercial application of bamboo in construction and other value-added industrial applications worldwide.

## 2. Materials and Methods

### 2.1. Materials

Giant bamboo (*Dendrocalamus asper* (Schult.) Backer) culms, approximately four years old, were collected from Sukabumi, West Java, Indonesia. The bamboo had an average density of 0.53 g cm^−3^. The bamboo strands were bonded using an industrial grade phenol-formaldehyde (PF) adhesive (PT Pamolite Adhesive Industry, Probolinggo, East Java, Indonesia). Paraffin was used in the adhesive mixture to improve the water resistance of the BOSB panel.

### 2.2. Characterization of Adhesives

The analysis of solid content began by depositing 1 g of adhesive onto aluminum foil and then subjecting it to a constant temperature of 103 ± 3 °C for 3 h in an oven (Memmert Celsius 10.0, Memmert, Germany). After the drying process was finished, the sample was moved to a desiccator for weighing. The solid content was determined by dividing the weight of the material after oven drying by its original weight. To determine the acidity level of the adhesive, a pH meter with an electrode probe was used. The pH reading was documented after inserting the probe into the specified container holding the adhesive sample.

The gelation time was measured using the Techne GT-6 gel time meter (Techne Incorporated, Burlington, NJ, USA). A water bath with dimethyl sulfoxide was used to reach a temperature of 135 °C for this purpose. The time taken for the adhesive to undergo gelation was meticulously noted and documented. Once the adhesive reached its maximum gelation time, the automatic timer stopped and displayed “gel” along with the corresponding numerical value on the screen.

The evaluation of viscosity, cohesion strength, relaxation modulus, and torque included adding around 20 mL of PF adhesive samples into a glass gauge (C-CC27, AntonPaar, Graz, Austria) and placing it on a rotational rheometer (RheolabQC, AntonPaar, Graz, Austria). Measurements were carried out using a cc-type spindle no. 27 at a speed of 150/s. Additionally, an examination of the basic rheological properties of the PF adhesive was conducted over temperatures ranging from 25 to 150 °C.

### 2.3. Preparation of Heat-Treated Bamboo Strands

**Figure 1:**
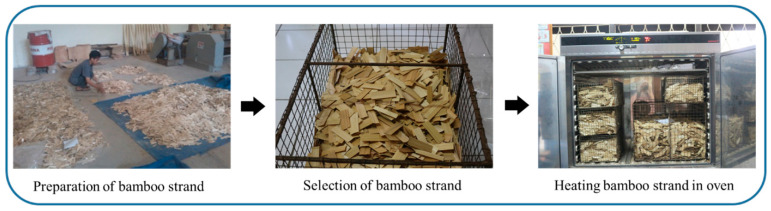
Heat treatment of bamboo strands used for manufacturing BOSB.

Figure 1 displays the illustration of heat treatment on bamboo strands. Bamboo culms were converted into strands (70 × 25 × 0.5 mm^3^). The selection of bamboo strands was conducted to ensure that they were free from fungi and other organisms. The bamboo strands were heated to 140 and 160 °C for 3 h in the oven. Apart from that, bamboo was also provided which was heated at a temperature of 160 °C for 1, 2, and 3 h. The strands were dried in the air for seven days before being dried in the oven for 36 h at temperatures ranging from 60–80 °C until the moisture content fell below 5%. The strand geometry was analyzed using Maloney’s approach [20], resulting in ratios of 3.23 ± 0.19 and 117.56 ± 11.15 units, respectively.

### 2.4. Characterization of Bamboo Strands

The chemical composition of untreated and pre-heat-treated strands, i.e., holocellulose, α-cellulose, hemicellulose, Klason lignin, and extractives content, was investigated in this research. The Browning technique [21] was used to determine holocellulose and α-cellulose content, whereas Dence’s (1992) approach was used to estimate lignin concentration. Methods for extracting extractives soluble in both cold and hot water were carried out following TAPPI T-207 cm-99 criteria [22]. The extractives soluble in ethanol-benzene were determined using the ASTM D-1107-96 reference technique [23]. The procedure in TAPPI T-212 om-02 was used to determine extractive solubility in a 1% NaOH solution [24].

### 2.5. Functional Groups and Crystallinity Analysis of Bamboo Strands

Fourier-transform infrared (FTIR) spectroscopy (SpectrumTwo, Perkin Elmer, Waltham, MA, USA) was utilized to examine the functional groups of both untreated and pre-heat-treated bamboo strands. The infrared spectrum was measured at 25 ± 2 °C and in the wave number range of 4000–400 cm^−1^. An X-ray diffractometer (XRD, Maxima, Shimadzu, Kyoto, Japan) was used to investigate the crystalline characteristics of bamboo strands. Segal’s method was used to calculate the relative crystallinity [25,26].

### 2.6. Fabrication of Bamboo Oriented Strand Boards (BOSBs)

**Figure 2:**
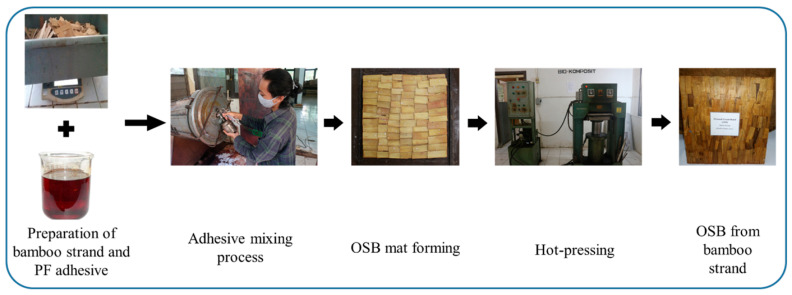
The process of manufacturing OSB utilizing bamboo strands.

Figure 2 depicts the process of manufacturing an OSB utilizing bamboo strands. Laboratory BOSBs with dimensions 300 mm × 300 mm × 9 mm and a target density of 0.7 g cm^−3^ were manufactured, following the technical procedure described by Maulana et al. [16]. Briefly, approximately 8% of PF resin mixed with 1% paraffin wax were sprayed onto the strands using a rotary blender. The BOSBs were then hot-pressed at a temperature of 135 °C for 9 min under a specific pressure of 2.45 MPa. After the hot pressing, the laboratory-made BOSBs were conditioned for seven days at a temperature of 20 ± 2 °C and a relative humidity of 65%. Each treatment was conducted in three replications to ensure accuracy. 

### 2.7. Physical and Mechanical Properties of BOSB

Both physical and mechanical properties of the BOSB were assessed in accordance with the provisions specified in the Japanese Industrial Standard JIS A 5908 [27]. The evaluated physical property parameters included moisture content (MC), density, thickness swelling (TS), and water absorption (WA). The mechanical property parameters consisted of modulus of elasticity parallel (MOE //) and perpendicular (MOE ⊥) to the face layer of BOSB, modulus of rupture parallel (MOR //) and perpendicular (MOR ⊥) to the face layer of BOSB, and internal bond (IB) strength. Afterwards, the obtained results were compared to the specifications of Grade O-1 commercial OSB panels according to CSA 0437.0 standard requirements [28].

### 2.8. Statistical Analysis

The study utilized two different research methods: investigating the effects of varying pre-heat treatment temperatures and examining the results of different pre-heat treatment durations. A consistent simple randomized design was used throughout the study in both trials. The influence of pre-heat treatment temperature was assessed at two specific levels: 140 and 160 °C for 3 h each. Similarly, the impacts of pre-heat treatment duration were explored across three time periods: 1, 2, and 3 h all heated at 160 °C. Data analysis involved ANOVA, and significant differences between variables were identified using Duncan’s Multiple Range Test (DMRT).

## 3. Results and Discussion

### 3.1. Adhesives Properties

Table 1 presents the essential features of the PF adhesive employed to attach the laboratory-made BOSB. The average solid content of the glue was 43.1%, which represents the proportion of non-volatile components. Increased solid content typically leads to enhanced bonding strength, as it promotes greater interaction with the wood substrate [29,30]. The resin content of this material has an impact on its viscosity, specific gravity, and gelation time. Generally, a higher resin concentration leads to increased viscosity and specific gravity, as well as a longer gelation duration.

The PF adhesive took 12 min and 24 s to undergo gelation, which is the process of forming a gel. Reducing the gelation period can shorten the duration of hot pressing in production, but it may also result in a decrease in the shelf life. The optimal gelation period for a PF adhesive, as specified by the SNI 06-4567 standards [31], is in the 30–60 min range.

**Figure 3:**
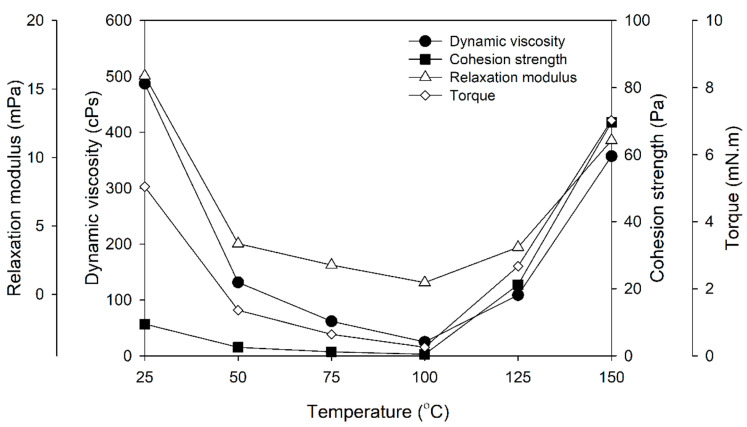
Basic rheological properties of PF adhesives as a function of temperature.

Figure 3 illustrates the fundamental rheological properties of PF adhesive, including dynamic viscosity, cohesion strength, relaxation modulus, and torque, in relation to temperature. These qualities are essential for the adhesive’s performance during application and bonding. At a temperature of 25 °C, the PF adhesive displayed an initial viscosity of 487.5 cPs, which decreased to 25.0 cPs at 100 °C. Subsequently, the viscosity increased to 357.4 cPs at 150 °C, at which point the adhesive underwent gelation at 135 °C. Cohesion strength also rose with temperature, from 9.5 Pa at 25 °C to 69.7 Pa at 150 °C, indicating greater molecular entanglement at higher temperatures.

During curing, the relaxation modulus of PF adhesive rose after 150 °C due to crosslinking of polymer chains. Viscosity impacts the torque needed for mixing or dispensing, with higher viscosity requiring greater force. As illustrated in Figure 3, temperature fluctuations influence adhesive viscosity and, consequently, the torque needed for mixing.

### 3.2. Chemical Composition

Pre-heat treatments, at 140 and 160 °C for 3 h, influenced the bamboo strands’ structural and chemical composition. The holocellulose content of bamboo strands heat-treated at 140 and 160 °C for 3 h decreased slightly compared to untreated bamboo strands, as shown in Table 2. The statistical study demonstrated that exposure to pre-heat treatment temperatures of 140 and 160 °C for 3 h did not result in significant changes (*p* > 0.05) in the holocellulose content. On the other hand, the hemicellulose content of bamboo strands decreased considerably from 28.82% in untreated bamboo strands to 24.07 and 20.30% due to pre-heat treatment at 140 and 160 °C, respectively, for 3 h. As a result of the decrease in hemicellulose content, the relative percentage of α-cellulose to other components probably increased. The statistical analysis revealed a significant impact (*p* < 0.01) of pre-heat treatment at temperatures of 140 and 160 °C for a duration of 3 h on the levels of hemicellulose and α-cellulose content. A previous study showed that hemicellulose was a chemical component easily degraded by heat treatment even at 126 °C [4,10,32]. Hemicellulose, being a polysaccharide characterized by a low degree of polymerization, is readily broken down when subjected to heat [33,34].

The proportion of Klason lignin increased from 27.98% (untreated) to 29.18% and 32.97% as a result of subjecting it to pre-heat treatment at temperatures of 140 and 160 °C, respectively, for a duration of 3 h. The statistical analysis revealed that subjecting bamboo strands to pre-heat treatment at 160 °C for 3 h had a significant (*p* < 0.05) impact on increasing the lignin proportion, in comparison to pre-heat treatment at 140 °C for 3 h. This might be attributed to the reduction in the levels of other polysaccharide components that have been degraded as a result of the pre-heat treatment. One possible cause of the increase in the Klason lignin content is the degradation of acid-soluble lignin. Zhang et al. [35] reported that heat treatment depolymerized the syringyl group of lignin, affecting its chemical and physical properties.

All extractives were reduced in each solvent as the treatment temperature increased. The lowest amount of extractive compounds was detected in each solubility after pre-heating at 160 °C for 3 h, except those dissolved in cold water (see Table 2). Pre-heat treatment had a substantial (*p* < 0.05) influence on extractive ingredient solubility, except in cold water. A previous study found that heating bamboo strands with steam at 126 °C for 1 h reduced their extractive component concentration [7,11]. Pre-heating at 160 °C for 3 h is regarded as more effective in reducing extractive substance levels resulting in beneficial alterations to the chemical components of the bamboo strands, as shown by variations at both structural and non-structural levels.

Table 3 shows the effect of pre-heat treatment at 160 °C under various durations on the chemical composition of the bamboo strands. The duration of pre-heat treatment applied to the strands has a noticeable impact on chemical constituents, including holocellulose, α-cellulose, hemicellulose, Klason lignin, and extractives. The decrease in holocellulose content from 74.46% to 73.09% suggests that some breakdown of holocellulose constituents occurs when exposed to a temperature of 160 °C for varying durations. Markedly, there was an increase in α-cellulose content (from 45.64% to 52.79%), resulting from the breakdown of hemicellulose (from 28.82% to 20.30%). This could potentially enhance the strength of bamboo due to the superior mechanical properties associated with α-cellulose. Additional research is necessary to comprehensively understand the correlation between α-cellulose content and the strength of bamboo.

A marked increase in Klason’s lignin content (from 27.89% to 32.97%), which may be due to the breakdown of hemicellulose, may alter the properties of the bamboo. The right balance of lignin can affect strength favorably, but excessive amounts can result in stiffness and brittleness. Therefore, identifying an intermediate value for lignin content seems to be an interesting avenue for further research. In addition, a noticeable decrease of the soluble extractive substance content occurred. These findings may have a direct impact on the optimization of the bamboo production process for various commercial applications and the overall quality of bamboo-based products.

### 3.3. Functional Group Analysis of Heat-Treated Bamboo Strands

**Figure 4:**
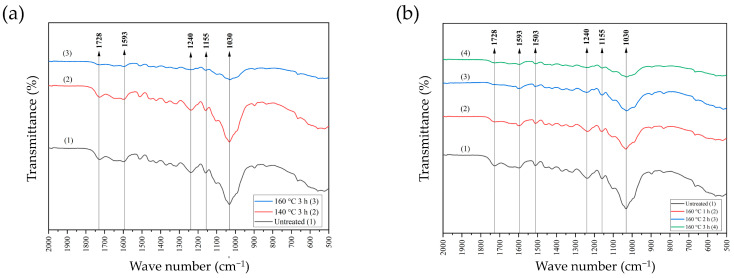
FTIR spectra of the bamboo strands after heat treatments at different conditions: (**a**) different temperatures, and (**b**) different durations.

Figure 4a displays the infrared profiles of untreated bamboo strands, bamboo strands treated at 140 °C for a duration of 3 h, and bamboo strands treated at 160 °C for a duration of 3 h. There are peaks at wave numbers 1030 cm^−1^, 1155 cm^−1^, 1240 cm^−1^, 1593 cm^−1^, and 1728 cm^−1^. No significant difference existed between the magnitude of the peaks found in untreated bamboo strands and bamboo strands pre-heat-treated at 140 °C for 3 h. The untreated bamboo strand had higher peak values compared to the sample pre-heat-treated at 160 °C for 3 h. The peak at 1593 cm^−1^ is associated with aromatic skeletal vibration with C=O stretch from lignin [36], and was substantially lowered after heat treatment. The peak at 1240 cm^−1^ reinforces the assumption of the existence of C=O from lignin [37], which was reduced owing to heat treatment, notably at 160 °C for 3 h. This phenomenon is presumably due to the reactivity of syringyl units derived from acid-soluble lignin (ASL), which heat treatment affects. According to the report of Maulana et al. (2021), syringyl units from lignin are more easily degraded by steam treatment than guaiacyl units. The peak at 1507 cm^−1^ is associated with aromatic skeletal vibration (C=C) [36], which proves the presence of a guaiacyl unit of lignin that has been stable against heat treatment up to 160 °C. The peak at 1728 cm^−1^ indicates the existence of C=O valence vibration from xylan in the hemicellulose [37,38,39], which is decreased by heat treatment at 160 °C for 3 h. The peak at 1155 cm^−1^ represents the C–O–C asymmetric valence vibration of cellulose and hemicellulose, which diminished owing to heat treatment at 160 °C for 3 h. The phenomenon of decreased polysaccharides, notably hemicellulose, is corroborated by a peak at 1030 cm^−1^, which shows the presence of C–O stretching of primary alcohol [36], which has diminished owing to heat treatment. Based on the results obtained, a temperature of 160 °C for 3 h more effectively reduced functional groups that can interfere with the BOSB manufacturing process.

The next step of this research was to evaluate the most effective and efficient pre-heat treatment duration to enhance the properties of the bamboo strand to be used as BOSB. Based on the results of spectra observations made on untreated bamboo strands and bamboo strands with 160 °C heat treatment for 1, 2, and 3 h, the same peaks were found as in Figure 4b, with a decreasing propensity as the heating duration increased. The reduction was found in the peaks with wave numbers 1030 cm^−1^ and 1728 cm^−1^, indicating the degradation of hemicellulose functional groups as the heat treatment duration increased. This phenomenon confirms hemicellulose degradation due to heat treatment (Table 3). Heat treatment easily degrades hemicellulose because it has an amorphous structure compared to other structural components [33,34].

### 3.4. Crystallinity of Heat-Treated Bamboo Strands

**Figure 5:**
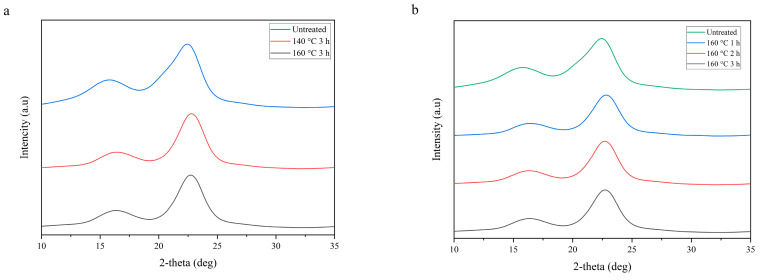
XRD graph of the bamboo strands after heat treatments at different conditions: (**a**) different temperatures, and (**b**) different durations.

The XRD spectra of the untreated and treated bamboo strands are shown in Figure 5a. All samples showed a typical cellulose XRD pattern with two prominent peaks, namely at 2θ of 18° and 22.5°, attributed to the amorphous and crystalline part of the cellulose, respectively. The crystallinity index (CI) can be calculated to investigate the crystalline structural changes due to the steaming treatment. The CI was defined as the degree of crystallinity calculated using Turley methods; the detailed empirical equation was readily available in previous literature [40,41]. The calculated CI of the untreated sample was found to be 41.5%. It significantly increased to 62.5% and 60.0% when the samples were heat treated at 140 and 160 °C, respectively, for 3 h. A similar result was observed for Figure 5b, with the CI index of the sample increased to 61.5%, 60.5%, and 60.0% after heat treatment at 160 °C for 1, 2, and 3 h, respectively. The pre-heat treatment of the sample increases the crystallinity of the sample. The amount of amorphous region of the sample (i.e., hemicellulose) was partly decreased during steam treatment due to its lower degradation temperatures compared to the other parts (i.e., cellulose and lignin), leading to the increase of CI [42]. Heat treatment of bamboo strand samples for 3 h at 140 °C increased the CI index by almost 1.5 times. This finding is in line with the hemicellulose content of the samples shown in Table 3. However, at higher steam temperature, i.e., 160 °C, increasing the duration of heat treatment led to a smaller increase in the CI index. This was probably due to the start of the crystalline part degradation [40]. Hence, finding the optimum temperature and treatment time was crucial to optimize the crystallinity degree of the heat-treated bamboo strands used for manufacturing BOSBs.

### 3.5. Properties of BOSB Panels

#### 3.5.1. Effects of Heat Treatment Temperature on BOSB Properties

**Figure 6:**
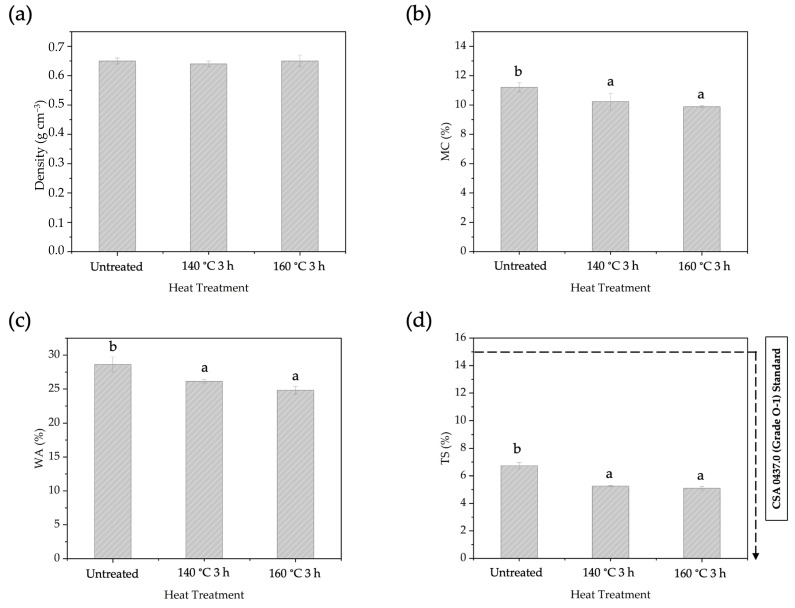
The density (**a**), MC (**b**), WA (**c**), and TS (**d**) of BOSBs fabricated from bamboo strands at different temperatures of pre-heat treatment. Different letters on the bar chart indicate differences of significance at *p* < 0.05 as determined by DMRT and ANOVA.

The physical properties of the BOSB after different temperatures of pre-heat treatment are presented in Figure 6. The density of the manufactured BOSBs ranged from 0.64 to 0.65 g cm^−3^ and can be classified as medium (0.6 to 0.79 g cm^−3^). The BOSBs manufactured with different heating procedures exhibited similar density values. This suggested that the density of BOSBs is homogenous. The MC values of BOSBs varied from 9.8% to 11.2%. In addition, the WA and TS values of OSBs ranged between 26.13% and 28.60%, and between 5.09% and 6.73%, respectively. All of the BOSB samples met the TS value requirements of the commercial standard CSA 0437.0 for OSBs. The statistical analysis indicated that the pre-heating temperature had a significant impact on MC, WA, and TS values. The DMRT analysis revealed that the MC, WA, and TS values of BOSBs produced from strands heat-treated at 160 °C for 3 h were significantly different compared to other samples. These results suggest that, as happens for wood after pre-heat treatment, particularly at a temperature of 160 °C for 3 h, the sample’s ability to absorb water was significantly decreased.

Increasing the temperature during the heat treatment enhanced the BOSB’s dimensional stability. This improvement is attributed to the changes in hemicellulose and extractive content that occur at elevated temperatures. The data presented in Table 2 illustrate that the heating temperature has an impact on the hemicellulose and extractive content of bamboo strands. Markedly, at 140 and 160 °C there was a decrease in hemicellulose content. It is well known that these components contain hydroxyl groups, which contribute to the moisture-absorbing properties of woody materials [43,44,45]. The decrease in hydroxyl groups can also be explained by the increase in CI. The heat treatment reduces the number of hydroxyl groups [46], leading to an increase in CI [47] and consequently reducing the hygroscopicity of bamboo strands. As a result, the reduction in hydroxyl groups through heat treatment can account for the stability observed in BOSB panels.

**Figure 7:**
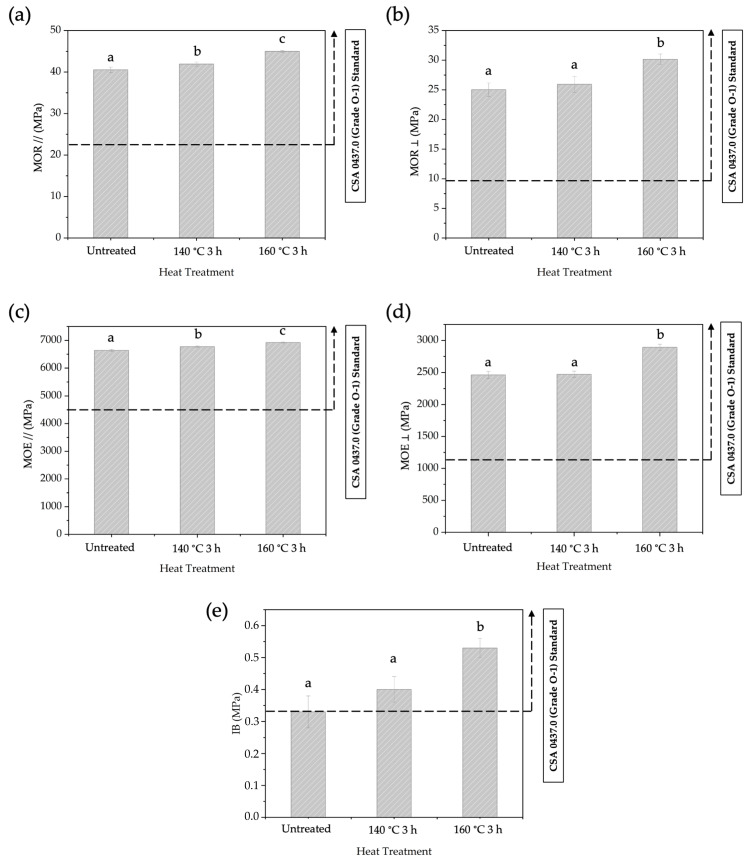
The MOR // (**a**), MOR ⊥ (**b**), The MOE // (**c**), MOE ⊥ (**d**), and IB (**e**) of BOSBs fabricated from bamboo strands at different temperatures of pre-heat treatment. Different letters on the bar chart indicate differences of significance at *p* < 0.05 as determined by DMRT and ANOVA.

The mechanical properties of BOSBs after different temperatures of pre-heat treatment are presented in Figure 7. The average values of MOR // and MOR ⊥ ranged from 40.54 to 44.99 MPa and 25.03 to 30.15 MPa, respectively. The average values of MOE // and ⊥ ranged from 6637 to 6920 MPa and 2462 to 2892 MPa, respectively. Additionally, the average values for IB strength ranged from 0.33 to 0.53 MPa. Statistical analysis indicated that there was an influence on the mechanical properties such as MOR, MOE, and IB due to varying pre-heat treatment temperatures. BOSB panels made from strands heat-treated at temperatures of 140 and 160 °C met the requirements of CSA Standard 0437.0 for mechanical properties. However, upon further statistical analysis, it was found that certain mechanical properties such as MOR ⊥, MOE ⊥, and IB showed no significant difference between untreated BOSBs and BOSBs made from strands pre-heat-treated at 140 °C for 3 h. Furthermore, the IB value of untreated BOSBs did not meet the requirements of the commercial standards. Consequently, it can be concluded that BOSBs manufactured using bamboo strands heat-treated at a temperature of 160 °C for 3 h exhibited higher performance. Comparative analysis through DMRT confirmed that this particular type of BOSB displayed significantly better mechanical properties compared to other BOSBs.

This research reveals that increasing the heat treatment temperature from 140 to 160 °C improved the mechanical properties of BOSBs. The improvement may be attributed to the removal of extractive substances during pre-heat treatment. In particular, Table 2 shows a significant reduction in extractives after pre-heating, notably at 160 °C for a period of 3 h. It is considered that this result has a positive influence on the bonding process. Previous study demonstrated that the extractive content interfered with the binding process during BOSB manufacture [8,10,20,48]. Bamboo commonly includes various extractives that might impact its physical qualities [49]. Pre-heat treatment at 160 °C, proved to reduce the levels of extractive substances (Table 2), makes the BOSB adhesion process more efficient and enhances the physical and mechanical properties of the BOSB. It thus explains the observed enhancement of the mechanical properties of the composites, especially the IB strength values of the BOSBs fabricated from bamboo strands pre-heated at 140 and 160 °C for 3 h. The increase in CI value might also lead to better bending performance of the composites in terms of enhanced MOE and MOR values.

#### 3.5.2. Effects of Heat Treatment Duration on BOSB Properties

**Figure 8:**
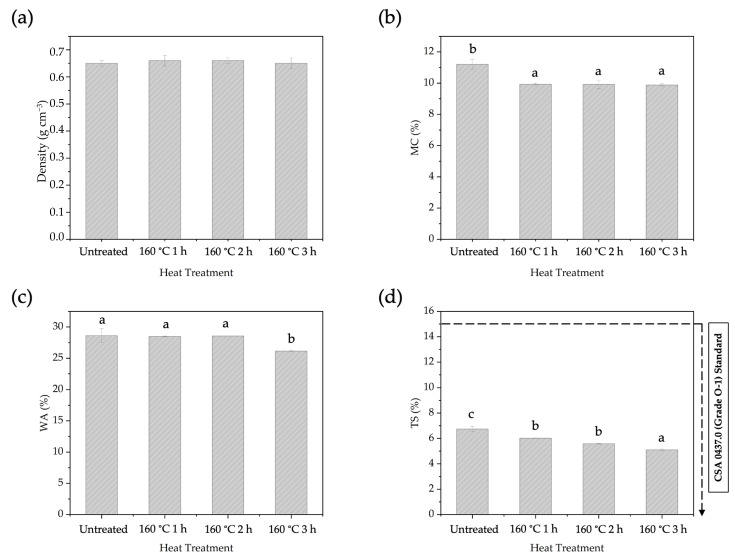
The density (**a**), MC (**b**), WA (**c**), and TS (**d**) of BOSBs fabricated from bamboo strands at different durations of pre-heat treatment. Different letters on the bar chart indicate differences of significance at *p* < 0.05 as determined by DMRT and ANOVA.

**Figure 9:**
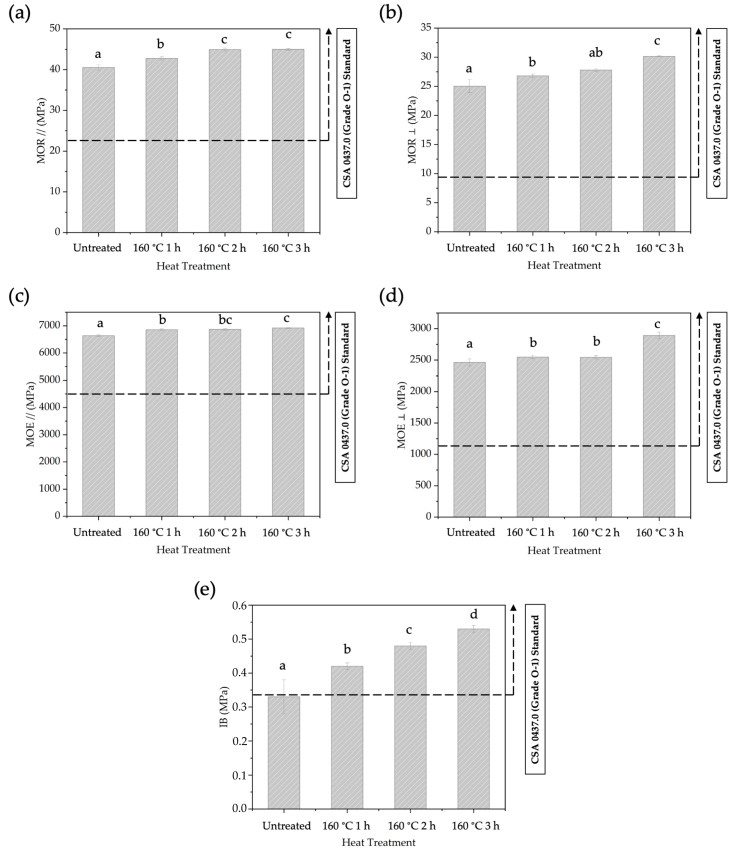
The MOR // (**a**), MOR ⊥ (**b**), The MOE // (**c**), MOE ⊥ (**d**), and IB (**e**) of BOSBs fabricated from bamboo strands at different durations of pre-heat treatment. Different letters on the bar chart indicate differences of significance at *p* < 0.05 as determined by DMRT and ANOVA.

The impact of different durations of pre-heating treatment on the physical and mechanical properties of BOSBs was investigated through the exposure of the strands to pre-heating at 160 °C (Figure 8 and Figure 9). The duration of the heating temperature had no effect on the density of the board. The MC fell significantly from 11.21% in BOSBs without pre-heat treatment to 9.88% in BOSBs subjected to 3 h of heating at the same temperature. The WA value also showed a consistent decrease from 28.61% to 26.15%, and TS value reduced from 6.74% to 5.10%. Statistical analysis shows that the duration of pre-heating has a significant influence on the MC, WA, and TS values, as does the pre-heating temperature. According to our research, extending the pre-heating treatment duration on bamboo strands led to increased dimensional stability of the BOSB. This phenomenon is also supported by DMRT results which show that BOSBs made with strands that were pre-heated for 3 h are different from others. However, the important finding in this case is that all BOSBs manufactured meet the requirements of the CSA 0437.O standard. This behavior may be ascribed to changes in the chemical makeup of the strand, notably a reduction in hemicellulose concentration. The experiment found that extending the pre-heat treatment period resulted in a considerable drop in hemicellulose content, from 28.82% to 20.30% (Table 3). This drop in hemicellulose leads to a decrease in the amount of hydroxyl groups present in the bamboo, thereby lowering the board’s ability to absorb water.

Heating the strands in BOSB production for up to 3 h at 160 °C led to substantial increases in MOR // and MOR ⊥ values. The highest values of MOR // and MOR⊥ were obtained at approximately 44.99 MPa and 30.15 MPa, respectively, as shown in Figure 9a,b. Correspondingly, the MOE // and MOE ⊥ have similar trends, with the highest values for both being 6920 MPa and 2892 MPa, respectively, as illustrated in Figure 9c,d. On the other side, the IB strength increased to 0.53 MPa (Figure 9e). Statistical analysis shows that the duration of pre-heating has a significant influence on the MOE, MOR, and IB values. Extending the pre-heat treatment duration to 3 h significantly enhances the mechanical properties of BOSBs. The BOSB made from strands pre-heated for 3 h showed the highest-quality mechanical properties among the evaluated samples. This observation is substantiated by the findings of the DMRT, which reveal that the mechanical properties of BOSBs formed from strands treated with 3 h of pre-heating are considerably different from those of the other treatment groups. This study demonstrates that all BOSBs manufactured from heat-treated bamboo strands meet the mechanical parameters specified by the CSA O437 standard [28].

This study revealed that the duration of the pre-heating process of bamboo strands at a temperature of 160 °C lasting from 1 to 3 h significantly influenced the physical and mechanical properties of BOSBs. Improved dimensional stability of BOSBs due to heating of the strands was indicated by decreased MC, WA, and TS values. Improved mechanical properties of BOSBs are shown by increased MOR, MOE, and IB. An interesting observation in this study is that the pre-heat treatment not only improved the properties but also significantly reduced their variation. In the context of effective and optimized BOSB manufacturing, it can be concluded that pretreatment at 160 °C for 1 h delivers good results, notably in terms of MOR, MOE, and IB, while still meeting CSA 0437.0 criteria.

This study indicated that the temperature and time of strand pre-heat treatment greatly influence strand properties, altering the physical and mechanical attributes of the resulting BOSB. Higher temperatures and longer durations considerably increase dimensional stability and quality of mechanical properties. These improvements are related to alterations in chemical components and crystal structure, as proven by functional group analysis and strand crystallinity (Figure 4 and Figure 5). Functional group analysis demonstrated a decrease in hemicellulose content due to heat treatment. Additionally, XRD research demonstrated that heat treatment enhanced the CI of bamboo strands, altering BOSB properties.

## 4. Conclusions

Pre-heating bamboo strands at temperatures of 140 and 160 °C, for 1 to 3 h, induced significant compositional changes in the bamboo through the reduction of extractive substances and hemicellulose. Furthermore, this alteration increased the proportion of α-cellulose and Klason lignin, as evidenced by the functional group analysis. Analysis of the crystallinity of bamboo strands indicated a rise in CI values following pre-heat treatment. These chemical transformations also affected the physical and mechanical properties of laboratory-made BOSBs, including their dimensional stability, and enhanced their MOE, MOR, and IB strength values. Enhancement in the physical and mechanical properties of BOSBs was observed with increasing heat treatment temperature up to 160 °C and heating duration up to 3 h. Markedly, all BOSBs manufactured from pre-heat-treated bamboo strands met the requirements of the CSA 0437.0 (Grade O-1) standard. A sufficient temperature and duration of pre-heat treatment of bamboo strands for manufacturing BOSBs with acceptable properties according to the CSA 0437.0 (Grade O-1) standard were 160 °C and 1 h, respectively. Overall, the results obtained suggest that the described pre-heat treatment technique can be employed for producing bamboo strands with enhanced properties, increasing their potential utilization in bamboo-based composite materials. However, future studies are needed to optimize the heat treatment process and test the long-term durability and performance of BOSBs. Furthermore, it would be useful to study the associated environmental impact involved with applying this method at an industrial scale.

## Figures and Tables

**Table 1:** Basic properties of PF adhesive used in this work.

Parameter	Unit	Value	Description
Solids content	%	43.10	at 135 °C
Gelation time	Minute	12.24	at 135 °C
pH	-	12.3	at 25 °C
Average viscosity	cPs	487.5	at 25 °C
Specific gravity	-	1.2	at 25 °C

**Table 2:** Chemical composition of bamboo strands after heat treatment for 3 h at different temperatures.

Chemical Components (%)	Heat Treatment *		
	Untreated	140 °C 3 h	160 °C 3 h
Holocellulose	74.46 ± 0.54	73.56 ± 0.34	73.09 ± 0.09
α-cellulose	45.64 ± 0.09 ^a^	49.48 ± 0.38 ^b^	52.79 ± 2.15 ^c^
Hemicellulose	28.82 ± 0.45 ^a^	24.07 ± 0.72 ^b^	20.30 ± 1.04 ^c^
Klason Lignin	27.98 ± 0.49 ^a^	29.18 ± 0.09 ^a^	32.97 ± 0.71 ^b^
Extractives solubility in:			
Cold water	5.25 ± 0.21	3.91 ± 0.34	4.35 ± 0.44
Hot water	7.31 ± 0.13 ^a^	6.55 ± 0.12 ^b^	5.44 ± 0.25 ^c^
1% NaOH	25.58 ± 0.46 ^a^	22.70 ± 0.74 ^b^	22.05 ± 0.37 ^b^
Ethanol benzene	7.87 ± 0.61 ^a^	5.56 ± 0.38 ^b^	5.34 ± 0.30 ^b^

* Statistical differences between treatments were identified through the use of different letters by DMRT at a significant level of 5%.

**Table 3:** Chemical composition of bamboo strands after heat treatment at 160 °C under different durations.

Chemical Components (%)	Heat Treatment *			
	Untreated	160 °C 1 h	160 °C 2 h	160 °C 3 h
Holocellulose	74.46 ± 0.54 ^a^	73.27 ± 0.36 ^b^	73.45 ± 0.04 ^b^	73.09 ± 0.09 ^b^
α-cellulose	45.64 ± 0.09 ^a^	48.54 ± 0.51 ^a^	52.12 ± 2.15 ^b^	52.79 ± 0.95 ^b^
Hemicellulose	28.82 ± 0.45 ^a^	24.74 ± 0.88 ^b^	21.33 ± 2.18 ^bc^	20.30 ± 1.04 ^c^
Klason Lignin	27.98 ± 0.49 ^a^	30.37 ± 0.38 ^b^	31.99 ± 0.08 ^c^	32.97 ± 0.71 ^c^
Extractives solubility in:				
Cold water	5.25 ± 0.21 ^a^	4.99 ± 0.17 ^ab^	4.89 ± 0.19 ^ab^	4.35 ± 0.44 ^b^
Hot water	7.31 ± 0.13 ^a^	7.50 ± 0.12 ^a^	6.82 ± 0.08 ^b^	5.44 ± 0.25 ^c^
1% NaOH	25.58 ± 0.46 ^a^	22.87 ± 0.25 ^b^	22.59 ± 0.10 ^b^	22.05 ± 0.37 ^b^
Ethanol benzene	7.87 ± 0.61 ^a^	6.91 ± 0.05 ^a^	5.50 ± 0.21 ^b^	5.34 ± 0.30 ^b^

* Statistical differences between treatments were identified using different letters (a,b,c) by DMRT at a significant level of 5%.

## Data Availability

The authors confirm that the data underlying the research are included in the article. The raw data that support the results are available upon reasonable request from the corresponding author.
